# Minimizing Surgically Induced Astigmatism at the Time of Cataract Surgery Using a Square Posterior Limbal Incision

**DOI:** 10.1155/2011/243170

**Published:** 2011-11-02

**Authors:** Paul Ernest, Warren Hill, Richard Potvin

**Affiliations:** ^1^TLC Eyecare & Laser Centers, 1116 W. Ganson, Jackson, MI 49202, USA; ^2^East Valley Ophthalmology, Mesa, AZ 85206, USA; ^3^Science in Vision, Burleson, TX 76028, USA

## Abstract

*Purpose*. To compare the surgically induced astigmatism from clear corneal and square posterior limbal incisions at the time of cataract surgery. *Methods*. Surgically induced astigmatism was calculated for a set of eyes after cataract surgery using a temporal 2.2 mm square posterior limbal incision. Results were compared to similar available data from surgeons using clear corneal incisions of similar size. *Results*. Preoperative corneal astigmatism averaged 1.0 D and was not significantly different between the incision types. Surgically induced astigmatism with the 2.2 mm posterior limbal incision averaged 0.25 ± 0.14 D, significantly lower in magnitude than the aggregate surgically induced astigmatism produced by the 2.2 mm clear corneal incision (0.68 ± 0.49 D). *Conclusion*. The 2.2 mm square posterior limbal incision induced significantly less, and significantly less variable, surgically induced astigmatism relative to a similar-sized clear corneal incision. This is likely to improve refractive outcomes, particularly important with regard to premium intraocular lenses.

## 1. Introduction

Sutureless clear corneal incisions are now arguably the most common incisions made to perform cataract surgery with phacoemulsification, replacing scleral tunnel and limbal incisions. There are significant differences in the effects of clear corneal and scleral or limbal incisions, related to anatomical and physiological differences in the respective structures where the incisions are made.

The cornea is comprised of a regular lamellar structure of collagen fibrils that stretch from limbus to limbus, arranged in a lattice formation; this is what provides the primary structural support of the cornea and accounts for its transparency. The cornea is also avascular. In contrast, at the limbus, this regular structure is no longer evident. Vascular arcades are present, providing a potential source of fibroblasts [1].

The differences in the healing effects of incisions at the limbus and the cornea have been previously discussed in the literature [2–4]. Limbal incisions appear to heal more quickly and are more resistant to deformation pressure than those in the cornea. Clear corneal incisions also appear to increase the likelihood of endophthalmitis, potentially for the reasons above [5]. In short, there are no disadvantages to a limbal incision in terms of surgical safety.

From a structural point of view it is known that incisions for cataract surgery will induce a flattening effect when made on (or near) the steep axis of the cornea. This is termed surgically induced astigmatism (SIA). This effect is positively correlated with incision size (larger incisions generating more astigmatism, all other things being equal) and location (scleral or limbal incision inducing less astigmatism than clear corneal), though for small incisions the effect of location appears less critical [6, 7]. Wound construction also appears to have an effect, with square incisions reported to affect astigmatism the least [8].

Data related to surgically induced astigmatism in the recent literature is primarily related to clear corneal incisions. The data reported here summarize the astigmatic changes produced with a small (2.2 mm) square posterior limbal incision. These results are compared to a sample of results from clear corneal incisions.

## 2. Patients and Methods

### 2.1. Patients

A retrospective patient chart review was conducted at one site (PE) for a study of toric and spherical IOLs [9]. Those eyes with preoperative and postoperative keratometry results were identified and the relevant data were extracted from the files. Eyes were excluded if they had irregular (nonorthogonal) corneal astigmatism or if they had previous corneal surgery. 

The retrospective review of data included no protected health information (PHI). This obviated the need for a specific informed consent or IRB approval for the data collection undertaken here. In addition, all patients sign an acknowledgement that their deidentified PHI data may be used for research purposes when they are seen in the practice. 

Comparative surgically induced astigmatism data were obtained from one of the authors (W. Hill) from a website specifically designed to collect preoperative and postoperative keratometry data for the purposes of calculating surgically induced astigmatism [10]. This site provides the necessary spreadsheet and instructions to surgeons for the calculation of SIA. An option allows any surgeon who uses the spreadsheet to upload their data to the website for the purposes of aggregate analysis. Again, no PHI was collected so informed consent was not required for this data set.

Results from PH and the aggregate data from WH were tabulated in Microsoft Excel and analyzed using STATISTICA statistical software [11]. Differences between groups were calculated with *t*-tests and analysis of variance (ANOVA) tests, with significance at *P* < 0.05.

### 2.2. Surgery

For the patients at one site (P. Ernest) the preoperative corneal astigmatism was measured using a manual keratometer. The data were corroborated with an automated keratometry reading and the automated keratometry feature of the IOLMaster (Carl Zeiss Meditec, Jena, Germany). The automated keratometry readings were repeated on all patients postoperatively, and the comparison to the preoperative automated keratometry was used to calculate SIA. This eliminated potential variability based on different technicians performing manual keratometry. Postoperative readings were all taken more than 6 months after surgery.

**Figure 1:**
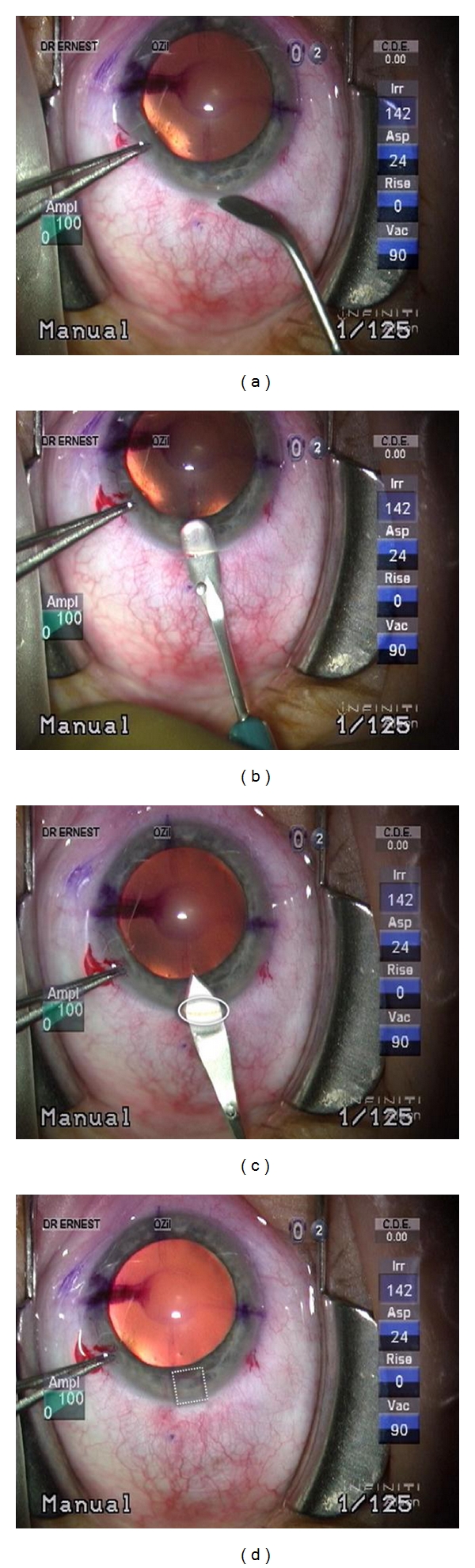
Construction of a posterior limbal incision.

The surgical technique employed in the time period in which the retrospective data were collected was constant for all patients. All incisions were temporal, 2.2 mm square posterior limbal incisions, using a technique previously described in the literature [12]. An illustration of the technique is shown in Figure 1. 

Surgical technique was not described in the aggregate SIA data collected off the web, but incision type (e.g., clear corneal, limbal) was generally indicated, and the size of the incision was noted. These variables were collected so that surgeons could submit different preoperative and postoperative data sets for different incision sizes and locations.

## 3. Results

The review of available clinical records from PE yielded a total of 38 eyes that both met the criteria for inclusion and had available postoperative keratometry data for analysis. Surgically induced astigmatism was calculated as the vector difference between preoperative and postoperative anterior corneal astigmatism as measured with automated keratometry. The SIA in this cohort averaged 0.25 D with a standard deviation of 0.14 D. 

A total of 1,712 eyes were available in the aggregate SIA data from W. Hill, from 51 different surgeons. The data were filtered to remove duplicates, include only incision sizes of 2.2 mm, and exclude those records where the incision type was not indicated. Where there were not at least 20 surgeries in any incision category (size and location) for a surgeon, those data were also deleted. After this data-screening step, 246 surgeries from 5 surgeons remained for comparative analysis. 

Average preoperative keratometry was not statistically significantly different between surgeons (*P* = 0.41); means ranged from 43.8 D to 44.5 D. The magnitude of preoperative corneal cylinder was also not statistically significantly different between surgeons (*P* = 0.13); means ranged from 0.79 D to 1.04 D.

Table 1 contains a summary of the surgically induced astigmatism by surgeon. Looking at the aggregate clear corneal incision data versus the Ernest posterior limbal data, there was a statistically significant difference in the surgically induced astigmatism by incision type (*P* < 0.001). Inside the clear corneal group, there was a statistically significant difference by surgeon (*P* = 0.003). As a post hoc test, the Ernest data were compared to the surgeon with the lowest average SIA from the clear corneal incision group (Surgeon 5); there was a statistically significantly lower mean SIA in the Ernest cohort (*P* = 0.001). Note, too, that the standard deviation of the Ernest cohort is less than half that of the cohorts of 4 of the 5 other surgeons, and 40% lower than Surgeon 5 despite having less than half the sample size.

**Figure 2:**
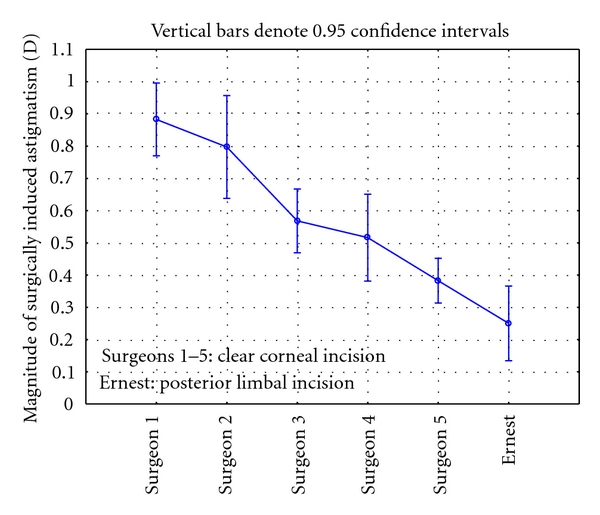
Surgically induced astigmatism with 2.2 mm incisions.

Figure 2 shows the calculated surgically induced astigmatism for the 5 surgeons. Surgeons 1 to 5 used 2.2 mm clear corneal incisions, while PE used a 2.2 mm square posterior limbal incision. There was a statistically significant difference in SIA by surgeon (*P* < 0.001). Post hoc testing showed that the Ernest data yielded a statistically significantly lower SIA than the other surgeons' data.

## 4. Discussion

The results suggest that the magnitude and the variability of surgically induced astigmatism with small incision surgery (2.2 mm) is significantly lower if a posterior limbal incision is used instead of a clear corneal incision. Results for the clear corneal incisions used here as comparative data are consistent with those recently reported in the literature. Wilczynski et al. reported SIA values of 0.50 ± 0.25 D with 1.7 mm and 1.8 mm surgical incisions [13]. Holland reported SIA values of 0.6 ± 0.4 D for 2.4 mm clear corneal incisions [14]. Masket reported that SIA results were somewhat lower with his 2.2 mm clear corneal incisions (0.35 ± 0.21 D) but it is worth noting that his incision was started with a groove at the temporal limbus [6].

Some of the variability of SIA data from surgeons other than Ernest is likely related to differences in technique between surgeons. In addition, intrasurgeon variability may be slightly higher if surgeons did not measure their postoperative keratometry 1 month or more after surgery, as there is some change in keratometry expected in that first month.

The minimization of the magnitude (and variability) of surgically induced astigmatism is important for modern day cataract surgery, particularly with the use of toric and multifocal intraocular lenses—lenses for which patients pay a premium, expecting premium vision. The success in effectively reducing astigmatism with toric lenses is affected by SIA [15]. Residual astigmatism after surgery will reduce the likelihood of spectacle independence for distance vision for these patients [16]. Minimizing astigmatism for multifocal IOLs is equally important, as even small amounts of residual astigmatism can compromise the outcome with regard to uncorrected visual acuity at distance [17].

Surgeons interested in reducing the magnitude and variability of induced astigmatism at the time of cataract surgery may want to consider the use of a 2.2 mm square posterior limbal incision.

## Figures and Tables

**Table 1:** Surgically induced astigmatism with 2.2 mm incisions.

Surgeon	Eyes	Incision type	Surgically induced astigmatism (D)		
			Mean	SD	Median
Surgeon 1	40	Clear corneal	0.88	0.60	0.72
Surgeon 2	20	Clear corneal	0.80	0.41	0.73
Surgeon 3	52	Clear corneal	0.57	0.41	0.41
Surgeon 4	28	Clear corneal	0.52	0.43	0.41
Surgeon 5	106	Clear corneal	0.38	0.23	0.34
*Ernest*	*38*	*Posterior limbal*	*0.25*	*0.14*	*0.26*
